# KneeMS to Monitor Knee Acoustic Emissions for Juvenile Idiopathic Arthritis

**DOI:** 10.1109/JTEHM.2026.3721498

**Published:** 2026-08-07

**Authors:** Ruchik Mishra, Quentin Goossens, Christopher Nichols, Ahmet Rasim Emirdagi, Chuoqi Chen, Onur Selim Kilic, Mohammad Nikbakht, Prabhkirat Bindra, Catherine Yang, Cate Garretson, Lori Ponder, Sampath Prahalad, Omer T. Inan

**Affiliations:** Georgia Institute of Technology Atlanta GA 30332 USA; Emory University Atlanta GA 30322 USA; School of Electrical and Computer EngineeringGeorgia Institute of Technology 1372 Atlanta GA 30332 USA; Coulter Department of Biomedical EngineeringGeorgia Institute of Technology Atlanta GA 30332 USA

**Keywords:** Juvenile idiopathic arthritis (JIA), knee acoustic emissions (KAEs), wearable sensing, machine learning, severity trend assessment, longitudinal analysis

## Abstract

Objective: Wearable sensing for capturing knee acoustic emissions (KAEs) can enable earlier detection and better management of juvenile idiopathic arthritis (JIA). In this paper, we expand on our previous work by validating the KneeMS wearable for individuals with JIA in a clinical setting against a benchtop system (Dytran).Methods and procedures: Acoustic features were recorded from the medial and lateral sides of the patellar tendon using both Dytran and KneeMS in 36 participants with JIA during flexion/extension (FE). We calculated the Spearman correlation coefficient ( *ρ *) between the acoustic features of both devices Acoustic features were recorded from the medial and lateral sides of the patellar tendon using both Dytran and KneeMS in 36 participants with JIA during flexion/extension (FE). We calculated the Spearman correlation coefficient between the acoustic features of both devices and assessed severity trends and longitudinal tracking of JIA knees from a machine learning (ML) pipeline differentiating active from inactive JIA knees.Results: Of 36 extracted features, six had the highest *ρ * (medial/lateral): RMS (0.79/0.70), MFCC1 (0.79/0.72), spectral slope (0.78/0.68), energy (0.78/0.67), Hjorth activity (0.75/0.71), and spectral flux (0.74/0.69). Our ML pipeline demonstrated an area under the receiver operating characteristic curve (AUC-ROC) mean of 0.77 ( *p< 0.05*) and showed a significant ( *p< 0.05*) upward trend in active knee prediction probabilities *P(A)* with clinical severity using KneeMS. For follow-ups, KneeMS *P(A)* moved in the clinically expected direction for 4 of 5 status-changing knees (versus 1 of 5 for Dytran); the clinical JADAS (cJADAS) score, available for most knees, was consistent with this finding.Conclusion: The high correlation between the acoustic features of Dytran and KneeMS, a significant AUC-ROC, significant JIA severity trend, and prospective longitudinal tracking make KneeMS a viable alternative to Dytran.Clinical impact: This validation establishes the foundation of home-based longitudinal joint assessment for JIA.Clinical and Translational Impact Statement: Wearable acoustic monitoring could enable at-home tracking of JIA disease activity, supporting timely treatment.

## I. Introduction

Juvenile Idiopathic Arthritis (JIA), considered an autoimmune disease, is a chronic form of arthritis that has a significant impact on an individual’s daily life [1], [2], [3], [4], [5], [6]. It is characterized by the onset of arthritis that occurs before the age of 16, with symptoms lasting for over six weeks [1], [7], [8]. JIA, as an umbrella term, encompasses the most common chronic condition in pediatric rheumatology [5], [9], [10], [11], [12], [13].

According to the International League of Associations for Rheumatology (ILAR), patients with JIA can be classified into seven mutually exclusive subtypes based on clinical features [5], [7]. Because no gold-standard diagnostic test exists, the diagnosis of JIA across all subtypes relies on clinical evaluation. This introduces subjectivity due to several factors, including clinicians’ experience levels and the heterogeneity of the population [1], [2]. Moreover, significant delays in the diagnostic and referral pathways to rheumatologists further compound the issue, leading to adverse long-term outcomes for individuals [14].

Several biomarkers have been identified that can aid in the diagnosis of JIA. Some of them involve an invasive approach to finding biomarkers, as summarized in [15], [16], and [17]. Two common blood-based laboratory biomarkers are the C-reactive protein (CRP) and erythrocyte sedimentation rate (ESR). However, these biomarkers are non-specific markers for inflammation, are not diagnostic of JIA, and can lead to false negatives [15], [18]. Hence, there is a need for stronger, non-invasive biomarkers for predicting joint health in JIA.

Consequently, to address the need for non-invasive, conclusive biomarkers to predict and monitor joint health, prior work has mainly focused on imaging modalities such as Doppler ultrasound and magnetic resonance imaging (MRI) to assess joint status [19], [20], [21], [22]. However, these methods lack longitudinal (serial measurements over longer periods of time as compared to spot check measurements only), continuous measurement capability and typically require costly, clinic-based equipment and trained operators, limiting their feasibility for long-term, everyday monitoring. Therefore, there is an unmet clinical need for objective, non-invasive tools capable of monitoring joint status longitudinally between clinic visits, particularly in pediatric populations where minimizing clinic burden is essential. As a solution to these limitations, researchers have focused on wearable sensing technologies to enable continuous, longitudinal monitoring of joint function during everyday activities [23], [24]. With this emerging class of non-invasive wearable sensors, electrical bioimpedance (EBI), has been used previously for JIA monitoring. Moise et al. [23] employed EBI to assess treatment effectiveness and to differentiate individuals with JIA from healthy controls. However, EBI provides an indirect, bulk measure of tissue properties and is typically obtained in quasi-static conditions, offering limited insight into how the affected joint behaves during functional movement [24], [25]. In contrast, knee acoustic emissions (KAEs) capture mechanical sounds generated by articular surfaces during motion, providing a local, movement-specific biomarker that can be continuously recorded using lightweight wearable sensors [26].

Initially, KAEs (and vibroarthrography) were proposed as a marker of joint health in adult inflammatory and degenerative arthritis [27], [28]. Richardson et al. [29] developed a multimodal sensing knee brace that integrates acoustic, bioimpedance, and additional sensing modalities to monitor patients with rheumatoid arthritis (RA) across multiple visits. Features from the brace recordings were used to predict disease activity scores and to estimate erythrocyte sedimentation rate (ESR), demonstrating that a wearable system can provide quantitative, longitudinal estimates of RA disease activity. In later work, Richardson et al. [30] evaluated the generalizability of KAE-based models across recording setups and populations, showing that models trained to predict inflammation-related markers in one cohort can maintain high performance when transferred to different hardware configurations (wearable versus benchtop accelerometers) and to independent osteoarthritis (OA) and preradiographic OA cohorts. These studies support KAEs as a robust, noninvasive indicator of joint health under varying practical conditions and motivate the development of KAE-based monitoring tools for pediatric arthritis.

In the context of juvenile idiopathic arthritis (JIA), Whittingslow et al. [31] first demonstrated that KAEs recorded during seated, unloaded (FE) cycles can be used to distinguish children with JIA from age- and sex-matched healthy controls. Using temporal and spectral features extracted from individual movement cycles and a logistic regression classifier, they achieved high subject-level classification accuracy and proposed a continuous joint health score that tracked clinical improvement at follow-up visits, thereby establishing KAEs as a digital biomarker of disease status in JIA.

Subsequent work has examined how task selection and loading conditions affect the diagnostic content of KAEs in JIA. Gharehbaghi et al. [32] compared acoustic emissions from both loaded and unloaded knee movements in children with JIA and healthy controls, showing that weight-bearing tasks can enhance the discriminative power of acoustic features for differentiating healthy, inactive, and active JIA knees. Building on these findings, Goossens et al. [33] conducted an extended clinical validation using benchtop accelerometers (Dytran) in a larger JIA cohort [34]. They extracted temporal, spectral, and cepstral features from seated FE cycles and demonstrated that a scalar “joint score” derived from these features differentiates healthy, inactive, and active JIA knees, further supporting KAEs as a clinically relevant biomarker of articular health.

Advanced signal processing approaches have also been proposed to capture the temporal structure of KAEs in JIA. For example, a preliminary change-point detection study based on the Teager energy operator investigated how abrupt transitions in the acoustic signal can be used to localize high-energy events within FE cycles and relate them to joint pathology in JIA [35]. This line of work complements feature-based analyses by focusing on time-localized characteristics of KAEs that may be sensitive to changes in cartilage contact mechanics and synovitis. However, existing JIA-focused KAE studies [31], [32], [33], [35] have relied on cabled benchtop sensing hardware and have neither addressed whether a low-cost wearable device can reproduce the relevant acoustic features, nor whether such a device can distinguish fine-grained JIA severity levels.

Despite this growing literature, several important gaps remain for the JIA population. Existing JIA KAE studies [31], [32], [33], [35] have been conducted using benchtop accelerometers and do not evaluate whether a low-cost wireless device with a small form-factor can reproduce key acoustic features or support practical, in-home monitoring. Moreover, prior work has primarily focused on distinguishing healthy from JIA-affected knees or active from inactive joints (ML model trained on active JIA and healthy controls with predcition on active vs inactive JIA), without systematically examining multilevel JIA severity (inactive, mild, moderate, severe) using multivariate statistical frameworks. In contrast, the present study directly validates a wearable knee acoustic system (KneeMS) against a Dytran benchtop accelerometer in children with JIA, quantifies feature-level agreement between the two devices, uses an ML pipeline to predict inactive vs active JIA while being trained on these two labels, and shows the first evidence that KneeMS predicted probabilities follow a significant trend with clinical JIA severity. In addition, descriptive statistics also show that the KneeMS’s *P(A)* moved in clinically relevant direction for four out of 5 follow-up knees that changed disease status bidirectionally from active to inactive or vice versa.

## II. Methodology

### A. Data Acquisition System

The acoustic system used in this study is based on a previously developed system, KneeMS [26]. The system includes the main board and the sensor board, which are illustrated in Fig. 1 (a). Two boards are connected through a flat flexible cable (FFC). An nRF52840 system-on-chip (SoC) (Nordic Semiconductor, Trondheim, Norway), a microUSB port, and two FFC connectors are on the front side of the printed circuit board (PCB). The micro Secure Digital (microSD) card connector is located on the back side of the main board. Regarding the sensor board, a tri-axial IIS3DWB accelerometer (STMicroelectronics, Geneva, Switzerland) is on the front side, while an FFC connector is on the back. The block diagram is shown in Fig. 1(b). The SoC communicates with the accelerometer by Serial Peripheral Interface (SPI). The data is saved locally on the microSD card. The accelerometer is configured to collect only the Z-axis data at 26.667 kHz. A customized graphic user interface (GUI) is developed to start the sensor and read data from the microSD card. The sensor can communicate with two sensor boards at the same time. To avoid folding the FFC and causing fatigue over the long term, we choose to keep one board connected to one sensor board.

**FIGURE 1.**
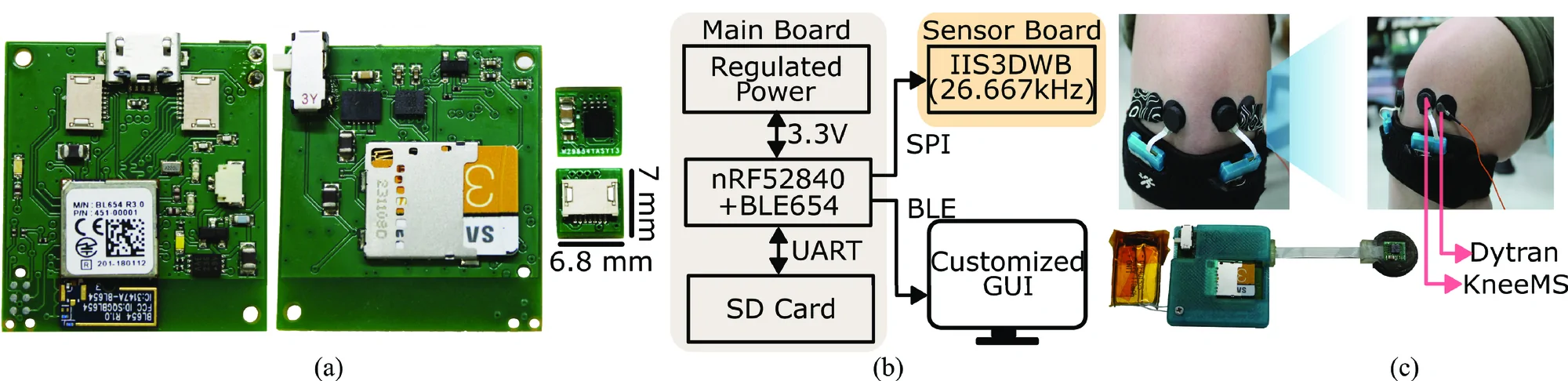
(a) Photos of the front and back sides of the main board and the mic board. The nRF52840, microUSB connector and the FFC connectors are located on the front side of the main board, while the SD card is positioned on the back. The IIS3DWB is located on the front side of the mic board. (b) A hardware block diagram of the KneeMS system. (c) The sensors setup for the Dytran sensor and the KneeMs system, as well as the photo of the whole KneeMS system.

The main board is secured in a customized case printed by a 3-D printer (Ultimaker 3, Utrecht, Netherlands). The sensor board is housed in a flexible silicone mold (Smooth-on, Macungie, PA) and is protected by a PLA holder (Ultimaker, Utrecht, Netherlands). The FFC cable is protected with a custom silicone protector (Smooth-on, Macungie, PA) to prevent it from detaching from the connectors. The main board features a 110 mAh Lithium-ion battery (Adafruit Industries, New York City, NY) and is held in the custom pocket of an off-the-shelf strap for ease and comfort during in-home wear. The sensor board is attached to the participants by medical-grade double-sided adhesive tape.

The benchtop model is a uniaxial accelerometer (Type 3225F7, Dytran Instruments, Inc., Chatsworth, CA) in combination with a data acquisition system (USB-4431, National Instruments, Austin, TX). Unlike the KneeMS, Dytran is bulky and not portable. As can be seen from Figure 1(c), the KneeMS and the Dytran were affixed to the skin using hypoallergenic double-sided adhesive pads (Rycote Stickies, Rycote Microphone Windshields Ltd, Stroud, UK). The Dytran accelerometer was further secured using latex-free kinesiology tape (OK TAPE PRO Kinesiology Tape, OK TAPE Inc.) applied over the microphones.

### B. Study Protocol

All data collected during this study were recorded at the Children’s Healthcare of Atlanta’s Center for Advanced Pediatrics. Prior approval was taken for this study (IRB # 2025P012814). Only children who had a prior diagnosis of JIA were included in this study. The demographic information of the recruited children is shown in Table 1. The demographics show a balanced participation between both male and female participants in the study. Follow-up visits for participants were also included in the dataset for both correlation and severity analyses.

**TABLE 1:** Participant demographics. Values are mean ± standard deviation. The number of unique knees is shown in parentheses.

	Overall	Female	Male
# Subjects (unique knees)	36 (68)	23 (44)	13 (24)
Age (years)	*12.9 ± 4.1*	*13.0 ± 4.2*	*12.8 ± 4.0*
Height (cm)	*153.3 ± 17.2*	*152.3 ± 15.7*	*155.1 ± 20.1*
Weight (kg)	*50.4 ± 21.0*	*47.9 ± 15.4*	*54.9 ± 28.5*

Acoustic data were collected from 36 participants (68 knees in total, data from the remaining 4 knees had either one of medial or lateral channels and hence were dropped) by placing both the Dytran and KneeMS sensors on the lateral and medial sides of the patellar tendon, as shown in Figure 1 (c). Each subject performed an FE task 10-12 times per leg, during which KAEs were recorded simultaneously by both Dytran and KneeMS. To maintain approximately the same speed, we showed each participant an instructional cartoon to imitate the FE exercise as described in [31], [32], with each FE cycle lasting about 4 seconds. In addition to collecting these acoustic signals, each knee was labeled as inactive (asymptomatic) or active (symptomatic) by a pediatric rheumatologist. The criteria for labeling a knee as active or inactive were adopted from previous work, where knees were considered ‘active’ if they had swelling alone, and/or tenderness and limited range of motion [33]. These labels were based on clinical exams conducted right before the recordings were taken. Each active knee was further classified as mild, moderate, or severe based on clinical exam by a pediatric rheumatologist. The clinician was asked to categorize each knee as Inactive, Active (mild), Active (moderate), or Active (severe) based on the degree of joint swelling, tenderness, and range-of-motion limitation observed during the examination. This knee-specific assessment differs from whole-body composite measures such as the Physician Global Assessment (PhGA), which evaluates overall disease activity across all joints [36]. One participant count has been repeated for both inactive and active (unilateral) categories since they were active on the first visit and inactive on the follow-up.

### C. Signal Preprocessing and Feature Extrac-tion

Figure 2 shows the signal processing and feature extraction pipeline. Due to the difference in sampling rates of Dytran (50 kHz) and the KneeMS (26.67 kHz), we resampled the Dytran signals to 26.67 kHz. At the beginning of each data collection session, we made a few taps near the participant’s patella to have large signal spikes. These spikes were used as a reference to synchronize the signals by aligning the first tap-induced spikes of both signals. All four channels per knee were synchronized: medial and lateral sides for both Dytran and KneeMS.

**FIGURE 2.**
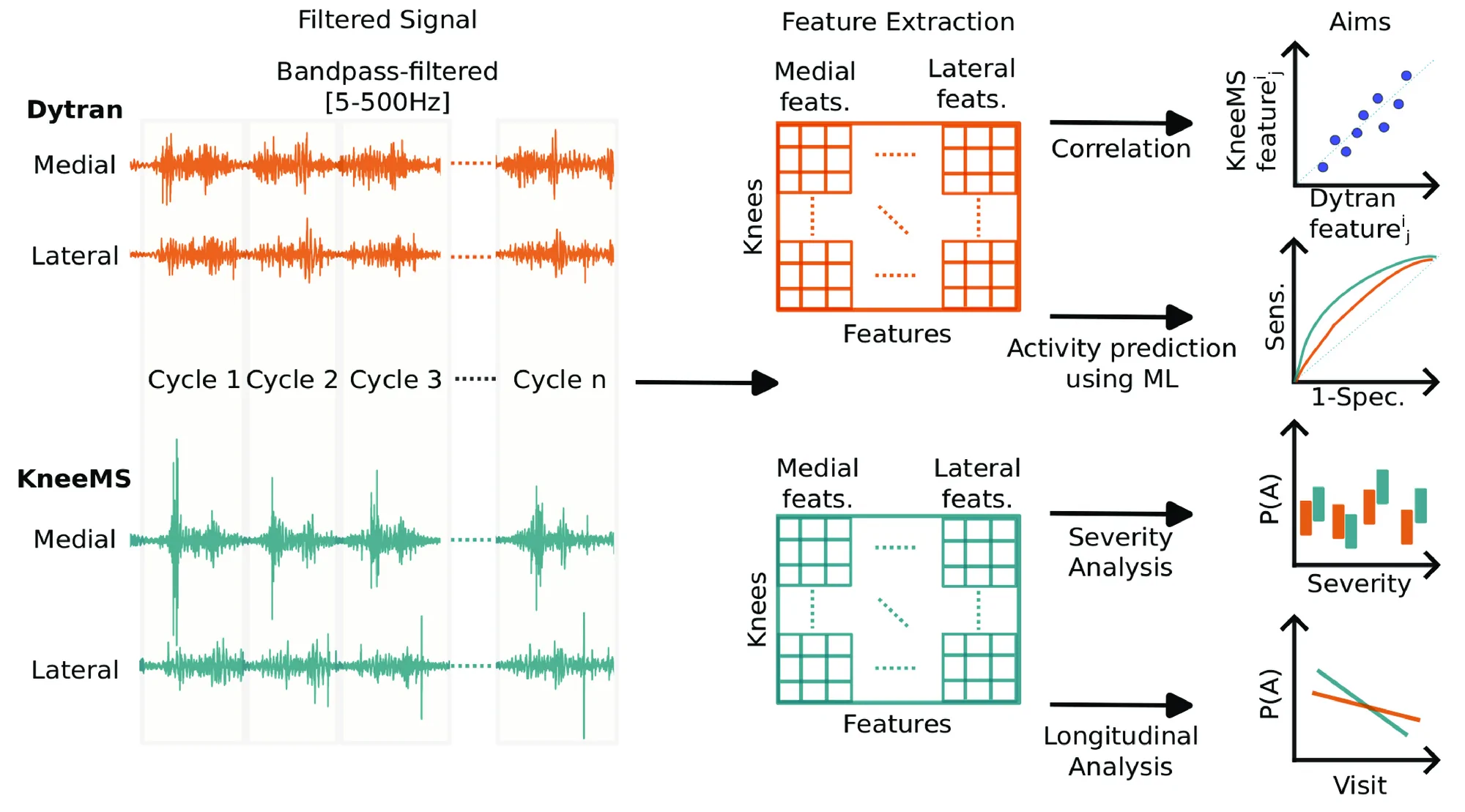
Signals from the medial and lateral sides of the patellar tendon is collected using Dytran and KneeMS and are synchronized after bandpass filtering between 5-500 Hz. Similar acoustic features are then obtained separately for each device and then analyzed across four aims: correlation, knee-level prediction using machine learning, JIA severity analysis, and longitudinal tracking of JIA knees to examine the change of active knee probability ( *P(A)*) across follow-up visits. In the correlation pipeline, *i, j* refer to the feature number and channel (medial or lateral), respectively.

Once the signals were aligned, they were bandpass filtered between 5 and 500 Hz. Since there were 10-24 FE cycles for each participant, we used only the data from these cycles and removed the signals between cycles. For each FE cycle, 50 ms window with 50% overlap was chosen (however, we experimented with different window and overlap lengths for our machine learning pipeline as shown in Supplementary materials Section S2) because knee acoustic emissions occur over a short time duration and appear as spike-like clicks in the time domain plot, as shown in Figure 2 [32], [37], [38].

Previous work on validating the KneeMS device against the Dytran used the RMS value of each FE cycle as the feature for comparison [26]. In addition, the authors used the KneeMS for a non-JIA population. In this paper, we expand on this feature set for the validation by including other temporal, spectral and mel-frequency cepstral coefficients [39]. The entire list of 36 features that we used for our correlation analysis between Dytran and KneeMS is shown in Table 3. These feature sets have been consistently used in previous work to make predictive analysis using machine learning for the JIA population [32], [37], [40].

**TABLE 2:** Number of severity observations by category. In.: inactive; A (mild/mod./s): active knees graded mild, moderate, or severe. For knees whose activity status changed between visits, each visit is counted as a separate observation; all other knees are averaged to a single observation.

	In.	A (mild)	A (mod.)	A (s)
# Total Knees	43	12	10	3
# Females	29	8	6	3
# Males	14	4	4	0

**TABLE 3:** Feature set computed per FE cycle for each signal channel (medial and lateral for both legs across both Dytran and KneeMS). Frame-based features use 50 ms windows with 25 ms overlap and are reported using six statistics: mean, median, std, IQR, min, max.

Domain	Feature family	Features
Temporal	RMS (frame-based)	RMS; mean, median, std, IQR, min, max.
	Waveform stats (cycle-level)	Peak-to-peak amplitude; skewness; kurtosis.
	Hjorth parameters (cycle-level)	Activity; mobility; complexity.
	TKEO (cycle-level)	Teager–Kaiser energy operator: mean; std; max.
Spectral	Spectral descriptors (frame-based)	Spectral centroid; spectral spread; spectral slope; spectral roll-off (90%); spectral flux; spectral entropy; zero-crossing rate (s^−1^); spectral crest; spectral skewness; spectral kurtosis; spectral flatness. For each: mean, median, std, IQR, min, max.
	Energy + sub-band energies (cycle-level)	Bandpower (10–1200 Hz). Sub-band energies: 10–100 Hz, 100–300 Hz, 300–600 Hz, 600–1200 Hz. Energy ratio: (300–1200 Hz)/(10–300 Hz).
	Spectrogram energy concentration (cycle-level)	STFT frame-energy concentration: $\max (E_{t})/\mathrm {mean}(E_{t})$, where $E_{t}=\sum _{f} |S(f,t)|^{2}$.
MFCC	MFCC_1..13_ (frame-based)	Coefficients 1–13; for each: mean, median, std, IQR, min, max.

### D. Correlation Between Dytran and KneeMS

After the feature extraction step, as shown in Figure 2, we find the most correlated acoustic features between Dytran and KneeMS using the Spearman correlation coefficient ( *ρ *). The reason for using these features is that they have been used previously to make predictions on knee health for various populations, including JIA [31], [32], [37], [38]. We have used Spearman’s *ρ * since it does not assume an underlying normal distribution to find monotonic relationships between variables.

Since for each knee, we have two sensor locations, each for Dytran and KneeMS (medial and lateral parts of the patellar tendon), we analyze the medial and the lateral sets of data separately to find Spearman’s *ρ *. The reason for treating the medial and lateral side separately is that the lateral side has a more impeded route compared to the medial side [37], [41], [42], [43]. For every sensor location, we average out each feature collected over a window per FE cycle, resulting in one value for each feature per cycle for correlation. Since every cycle of the Dytran and KneeMS is synchronized, we obtain an aligned feature set for both of the devices for comparison, i.e., for each Dytran feature per cycle, we have the corresponding KneeMS feature for that cycle.

### E. Machine Learning for JIA Predication and Severity Analysis

As previously discussed, one of the long-term goals of KneeMS is to extend beyond the clinical setting to in-home use for longitudinal monitoring of the knee. To accomplish this, we applied our ML approach to generate knee-level predictions using the KneeMS acoustic feature set in Table 3. For both KneeMS and Dytran-driven acoustic features, we used all 36 extracted features (see supplementary materials Section S1), consistent with our previous approach [44]. All features were z-score normalized using statistics computed from the training fold only and applied to the held-out data, to avoid data leakage. We used logistic regression (LR) to classify active versus inactive knees, trained separately for KneeMS and Dytran to enable a direct comparison between the two devices.

We used leave-one-subject-out cross-validation (LOSO-CV), in which all data from a given subject, both knees and any follow-up visit(s), was (were) placed entirely in either the training set or the test set (also across follow-up visits), but never split across both, thereby avoiding data leakage across dependent observations. To avoid overfitting, we used L2 regularization and performed hyperparameter tuning in an inner 3-fold CV loop to maximize the area under the receiver operating characteristic curve (AUC-ROC). Since there was an imbalance in the number of active versus inactive knees, class weights were set inversely proportional to class frequency.

For severity analysis, we use the same logistic regression pipeline, with the out-of-fold prediction probability (P(active)) of active knee. These severity labels (inactive, mild, moderate, and severe) were obtained by the board-certified pediatric rheumatologist during clinical examination. None of the severity labels were used during the training of our LR classifier. The left and the right knees were treated independently w.r.t labels since both knees could carry different severity and activity labels. These probabilities per knee, *P(A)* was tested against severity using Jonckheere–Terpstra test, which is a non-parametric, rank-based test used to test ordered trend across groups [45]. We assessed statistical significance using permutation across ‘N’ iterations to construct an empirical null distribution. This approach was used for both Dytran and KneeMS.

### F. Longitudinal Monitoring Using KneeMS

In this paper, we present the first set of exploratory results for longitudinal tracking using KneeMS. This analysis uses the same LR classifier approach. We trained the LR classifier on the knees of all subjects who had no follow-up visits, and the subjects with follow-up visits were held out as test subjects. This design allowed us to evaluate whether a model learned from independent single-visit observations could generalize to repeated measurements and capture clinically meaningful within-knee changes over time. Specifically, we examined whether the predicted probability of the active class, ( *P(A)*), derived from KneeMS acoustic features, changed in a direction consistent with the clinically determined change in activity status at follow-up—increasing when a knee became active and decreasing when it became inactive. This longitudinal capability is central to the long-term goal of using KneeMS as a wearable alternative to Dytran for at-home monitoring of JIA knees.

## III. Results and Discussion

### A. Spearman Correlation Between Dytran and KneeMS Features

Figure 3 shows the results for the top 6 highly correlated features between the Dytran and KneeMS for the medial side of the patellar tendon, combining FE cycles from right and left knees. Figure 4 shows the same for the lateral side for both the left and the right knee. Table 4 shows the high correlation between the acoustic features of the two devices with statistical significance ( *p < 0.001*). The 95% confidence intervals were computed by bootstrap resampling of knees (the independent unit of analysis; n = 68) with replacement over 5000 iterations, recomputing Spearman’s *ρ * on each resample.

**TABLE 4:** Knee-level Spearman correlation ( *ρ *) between KneeMS and Dytran acoustic features for the six most correlated features, with bootstrap 95% confidence intervals (resampling of *n=68* knees, 5000 iterations). All correlations were significant ( *p< 0.001*).

	Medial		Lateral	
Feature	*ρ *	95% CI	*ρ *	95% CI
RMS	0.79	[0.62, 0.92]	0.70	[0.51, 0.83]
MFCC-1	0.79	[0.65, 0.89]	0.72	[0.56, 0.84]
Spectral slope	0.78	[0.61, 0.90]	0.68	[0.49, 0.83]
Energy	0.78	[0.61, 0.90]	0.67	[0.48, 0.81]
Hjorth activity	0.75	[0.58, 0.88]	0.71	[0.52, 0.84]
Spectral flux	0.74	[0.57, 0.87]	0.69	[0.50, 0.83]

**FIGURE 3.**
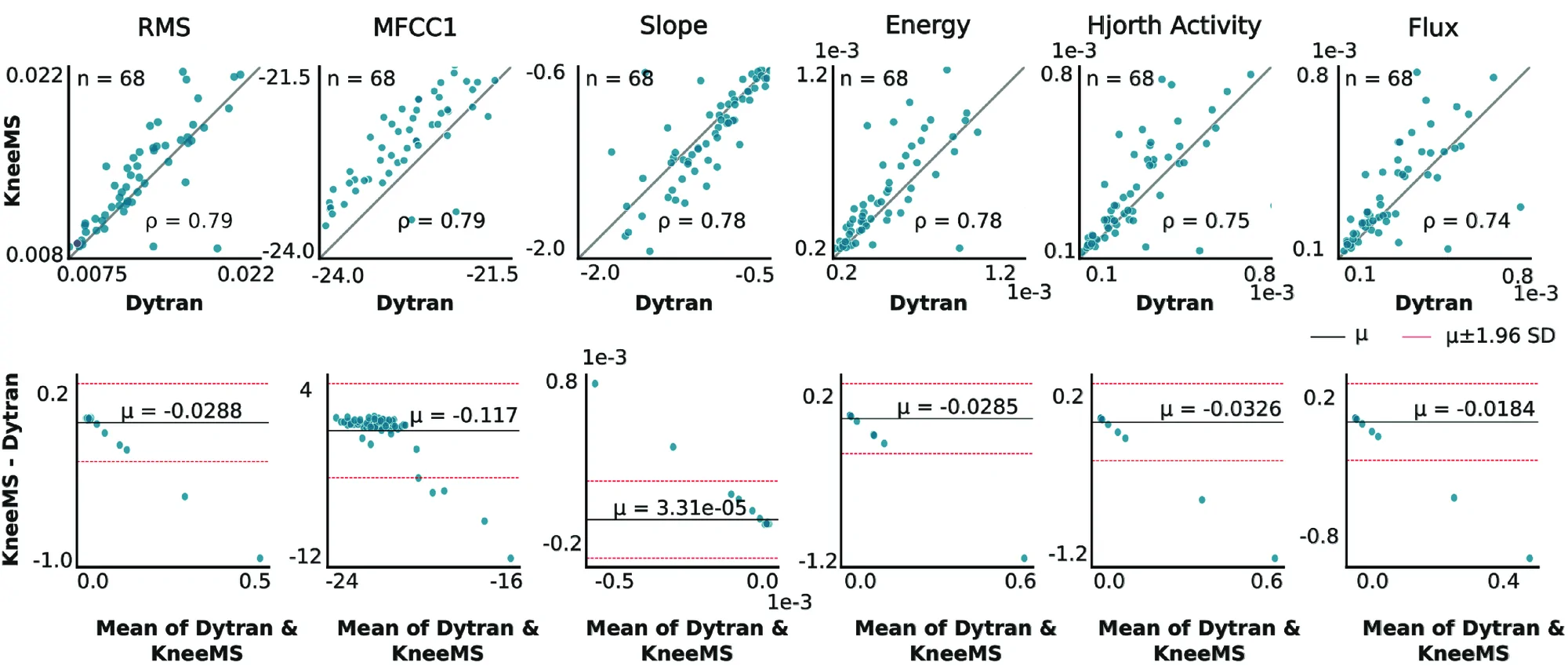
Row 1 shows the six most correlated features between Dytran and KneeMS (from the medial side) rms, MFCC1, spectral slope, energy, Hjorth activity cycle, and, flux out of the 36 features extracted. ‘n’ is the number of knees used to compare Dytran and KneeMS and is the Spearman Correlation Coefficient. Row 2: corresponding Bland–Altman plots (x = mean of the two devices, y = KneeMS - Dytran); the solid line ( *μ *) is the mean difference and the dashed lines are the ±1.96 SD limits of agreement.

**FIGURE 4.**
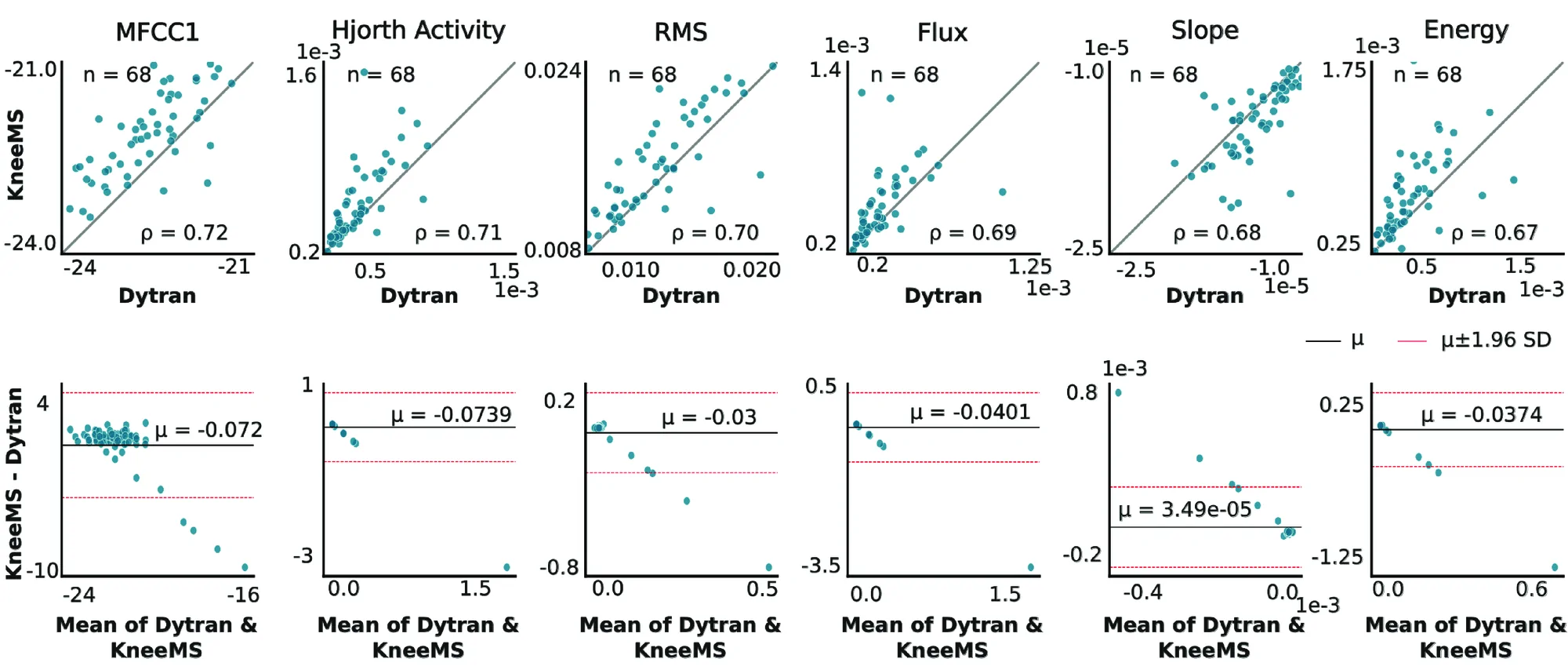
Row 1 shows the six most correlated features between Dytran and KneeMS (from the lateral side) RMS, MFCC1, spectral slope, energy, Hjorth activity cycle, and, flux out of the 36 features extracted. ‘n’ is the number of knees used to compare Dytran and KneeMS and is the Spearman Correlation Coefficient. Row 2: corresponding Bland–Altman plots (x = mean of the two devices, y = KneeMS - Dytran); the solid line ( *μ *) is the mean difference and the dashed lines are the ±1.96 SD limits of agreement.

This finding is consistent with previous research on KneeMS, where the RMS values of FE cycles were highly correlated across the two devices [26]. In addition to the RMS values, our extended results on other acoustic features too show that the KneeMS and Dytran have significantly similar acoustic properties. These six features have previously been shown to have predictive capabilities for monitoring knee health in the JIA population [31], [32], [33], [37], thereby making KneeMS a viable alternative to the benchtop gold standard for recording KAEs in the JIA population.

The high similarity between KneeMS and Dytran has several advantages, as were highlighted in previous work too: 1) KneeMS is almost 25 times lower in cost compared to Dytran, 2) KneeMS is a wireless system that is easily portable because of its lightweight and small form factor, 3) It can serve as a monitoring system for longitudinal knee health for patients with JIA.

### B. Machine Learning for Knee-Level JIA Disease Activity Prediction

Our ML approach, using an LR classifier with regularization and class weighting, was able to differentiate between active and inactive JIA knees (while trained on the same labels with LOSO-CV) with an AUC-ROC of 0.77, which significantly exceeded chance by permutation testing ( *p*-value = 0.005) (see Figure 5 (a)). In contrast, the LR-based classifier with Dytran’s acoustic features did not significantly exceed chance (AUC-ROC = 0.64; *p*-value = 0.063) (see Figure 5). To quantify the uncertainty in the classification performance for both the KneeMS and Dytran-based LR pipelines, we computed 95% Confidence intervals using subject-cluster bootstrapping across 2000 bootstrap resamples that were resampled with replacement. The out-of-fold AUC-ROC and AUPRC were reported as shown in Table 5. We did a hyperparameter sweep across four values of C (1/ *λ *) = [ *0.01,0.1,1,10*], which was chosen through an inner 3-fold cross-validation loop using only the training data in each fold.

**FIGURE 5.**
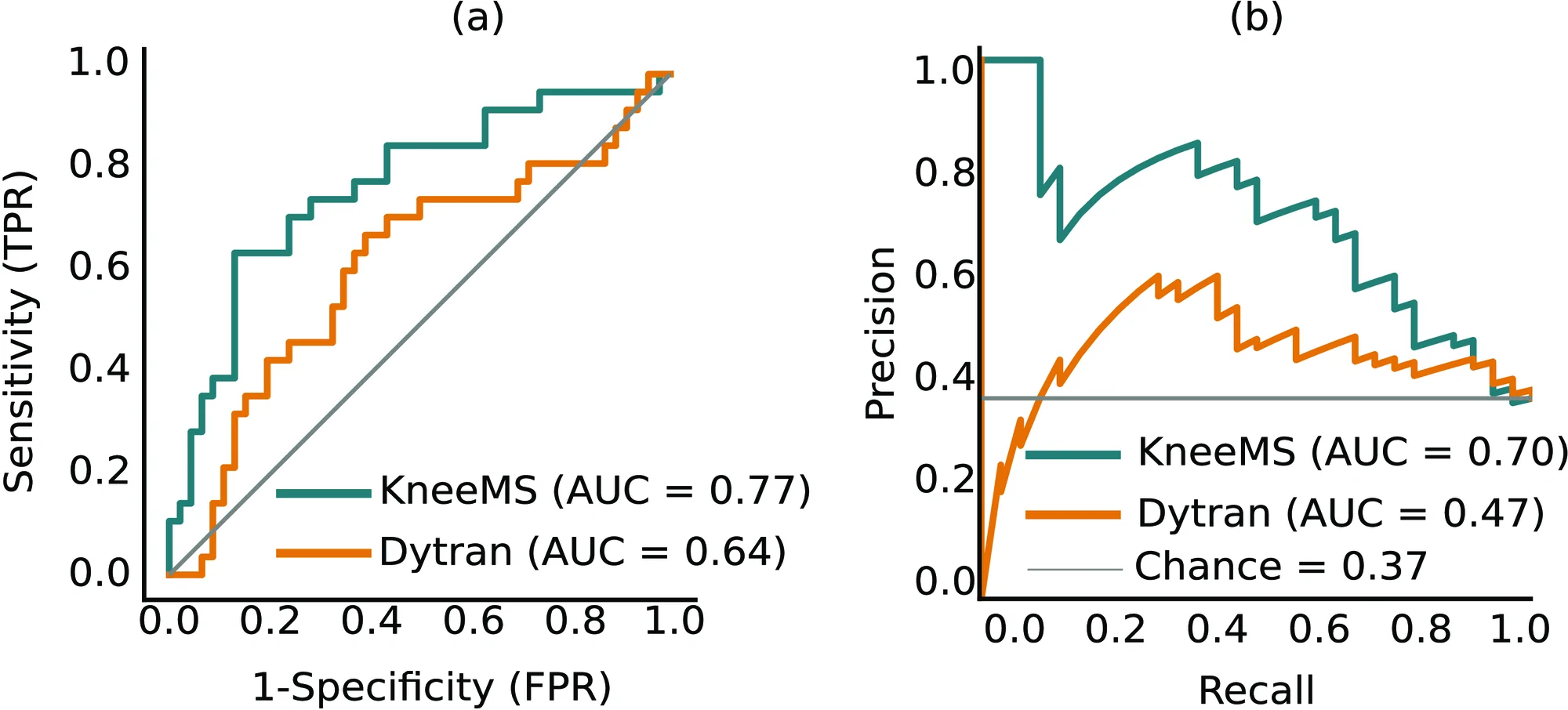
Performance of the LR pipeline for knee-level prediction of active vs inactive JIA knees. (a) AUC-ROC for both Dytran and KneeMS, (b) Precision-recall curve comparing Dytran and KneeMS.

Figure 5 and Table 5 show the performance of the LR classifier for both KneeMS and Dytran. KneeMS-based acoustic features perform better in classifying a JIA knee as active or inactive on all the performance metrics used making it a clear choice for knee-level JIA activity prediction. The confusion matrix with the exact number of samples has been shown in the Supplementary materials section S4.

**TABLE 5:** Comparing ML results for KneeMS and Dytran-based acoustic features.

Device	AUC-ROC	95% CI	AUPRC	95% CI	Sensitivity	Specificity	Balanced Acc.
KneeMS	0.77	[0.63, 0.88]	0.70	[0.48, 0.87]	0.679	0.809	0.74
Dytran	0.64	[0.52, 0.75]	0.47	[0.34, 0.66]	0.46	0.723	0.59

### C. JIA Severity Differentiation

As already discussed, one of the long-term goals of using the KneeMS is to provide in-home knee monitoring. A key application is to understand the difference between inactive and active knees. This is useful for tracking whether the medication or treatment protocol is effective over time for the patient with JIA. This would not only provide prior data to the rheumatologist about the knee’s health status but would also help mitigate the consequences of delayed referrals to rheumatologists.

In that aspect, it becomes crucial to find if KAEs recorded through KneeMS are capable of differentiating when a knee has reduced activity of inflammation and vice versa. Figure 6 shows the severity trend based on the probability of active knee ( *P(A)*) for out-of-fold knees using LR classifier, for each severity type for both KneeMS and Dytran. This severity trend was tested using the Jonckheere–Terpstra test where the out-of-fold KneeMS and Dytran prediction probabilities showed a significant increasing trend with severity grade (KneeMS: *ρ =0.38*, *p*-value < 0.001, Dytran: *ρ =0.20*, p = 0.047)(see Figure 6).

**FIGURE 6.**
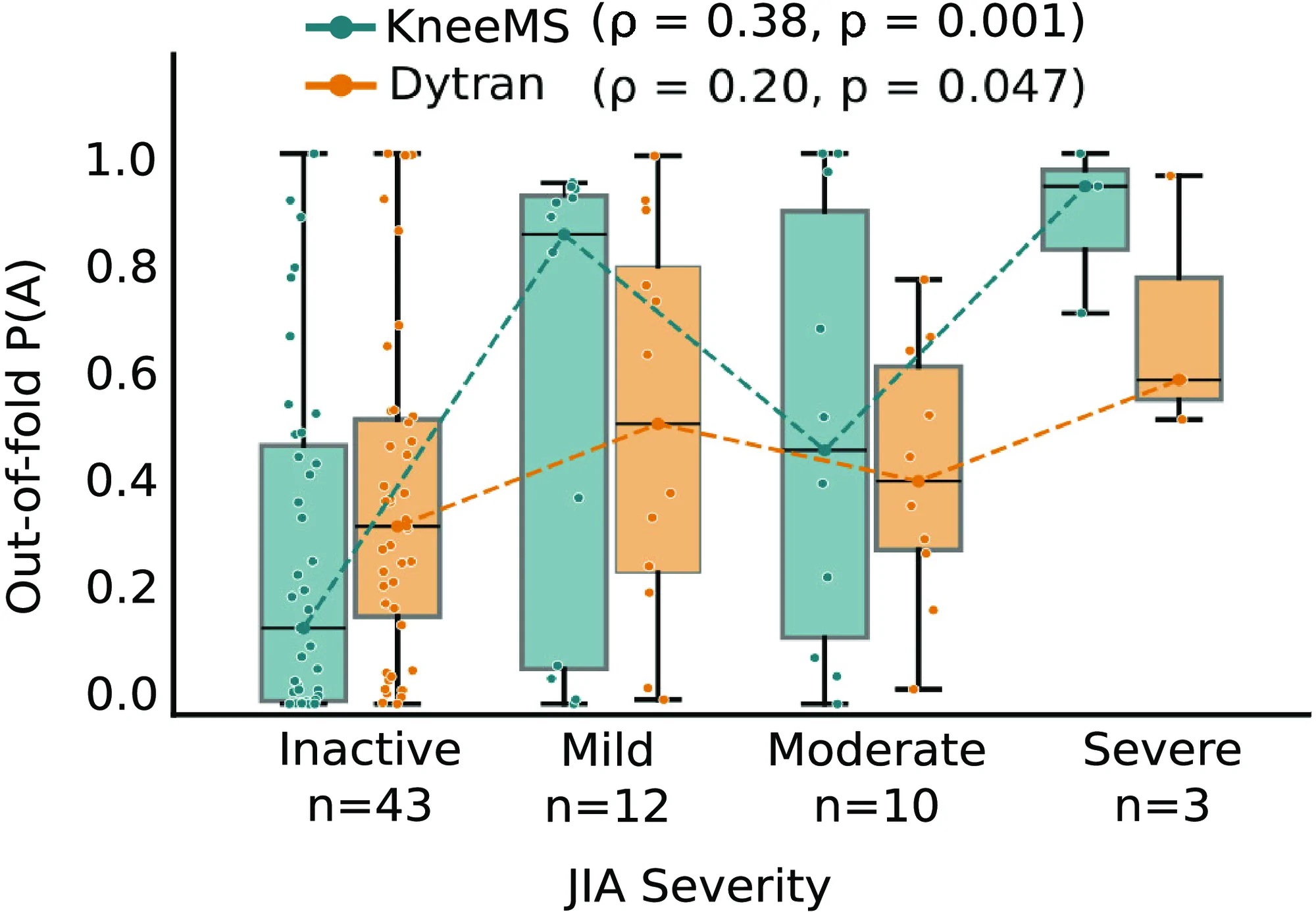
JIA severity trend w.r.t. KneeMS and Dytran prediction probabilities.

An important point to note here is that the overall trend for the KneeMS severity was driven mainly by the separation between active and inactive knees, where *P(A)* of inactive knees were significantly lower compared to the active knees. However, when the Jonckheere–Terpstra test was restricted to active knees only, the trend still remained in the same direction but was not statistically significant ( *ρ = 0.28*, *p*-value = 0.13). This observation is consistent given the smaller number of knees for higher severity grades like ‘severe’, hence limiting the statistical power for the severity test.

### D. Longitudinal Analysis

As an exploratory analysis, we conducted longitudinal analysis on 5 follow-up visits across 4 subjects. For this, we used the LR classifier and tested it on a held-out set of all the knees that had at least one follow-up, while the training data did not include any subject knees that had follow-ups. This out-of-fold prediction probability of active class *P(A)* was then tracked for the base and the follow-up visit(s).

*P(A)* for KneeMS-based predictions successfully moved in the clinically relevant direction for 4 out of 5 knees of subjects that had follow-ups, while Dytran’s *P(A)* moved in the clinically relevant direction for 1 out of 5 follow-ups (see Figure 7). Except for Subject 2’s (subject IDs are not universal) left knee, KneeMS *P(A)* moved in the direction of the JIA activity transition, including across multiple follow-up visits (e.g., Subject 3’s left knee, tracked correctly across three visits). In addition to this, KneeMS *P(A)* also moved bidirectionally, i.e., moving accordingly for both active-to-inactive and inactive-to-active transitions. For each visit-to-visit transition, we report *Δ P(A)* (see Table 6), which is the change in the predicted probability of the active class between consecutive visits ( $P(A) _{\textrm {current}}$ - $P(A) _{\textrm {previous}}$). So, a positive value of *P(A)* means the predicted value of active knee went up and vice-versa.

**TABLE 6:** Change in *P(A)* for KneeMS and Dytran across consecutive follow-up visits. A: active, I: inactive. Each row is one visit-to-visit transition.

Knee	Trans.	Visits	$\boldsymbol {\Delta P(A)_{\textbf {KneeMS}}}$	$\boldsymbol {\Delta P(A)_{\textbf {Dytran}}}$
Sub 1, R	I *→ *A	1–2	0.45	−0.39
Sub 2, L	A *→ *I	1–2	0.03	0.26
Sub 3, R	A *→ *I	1–2	−0.19	−0.71
	I *→ *I	2–3	−0.24	−0.26
Sub 3, L	I *→ *A	1–2	0.17	−0.07
	A *→ *I	2–3	−0.61	0.04
Sub 4, L	A *→ *I	1–2	−0.57	0.08

**FIGURE 7.**
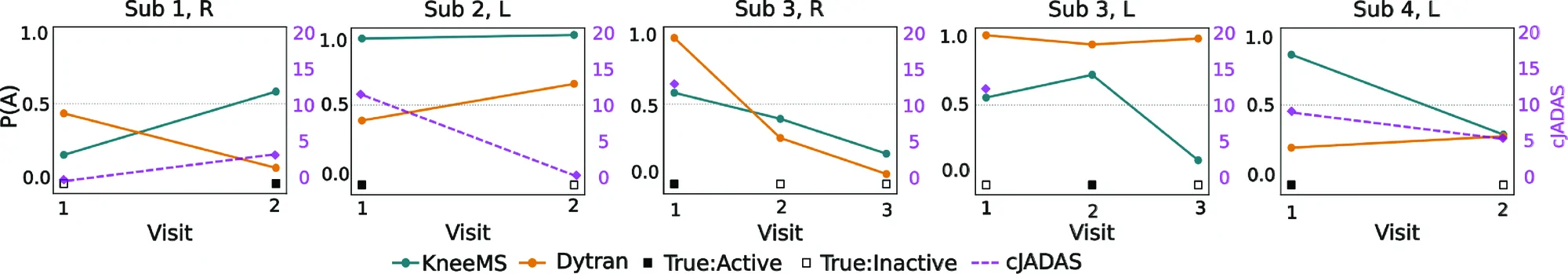
Prediction probabilities of Dytran and KneeMS across baseline and follow-up visits and comparison with cJADAS.

As an additional corroboration, we report the available cJADAS scores, a composite disease activity score available for most of the status changing follow-up visits (see Figure 7) [46]. From Figure 7, it can be clearly inferred for Sub 1’s right knee and Subject 4’s left knee, the disease activity status was consistent with the drop in cJADAS score for w.r.t. KneeMS’s *P(A)*.

Since we report this with only 5 follow-ups, the longitudinal analysis is descriptive as the sample size does not support any statistical analysis. However, these results show a prospective use of KneeMS for longitudinal monitoring of change of disease state for JIA knees.

### E. Clinical Impact

Current JIA management relies on periodic clinical visits, typically spaced months apart. A major reason is due to the shortage of pediatric rheumatologists and current long wait times for clinic appointments [47]. So, disease flares go undetected in-between clinical visits which lead to irreversible damage to the joints. Furthermore, clinical joint examination is inherently subjective, with variability across clinicians in detecting subtle changes in joint inflammation. A validated wearable acoustic monitoring device, such as KneeMS, could address these limitations by enabling objective, longitudinal assessment of joint status between clinic visits.

The present study represents the second and third stage of the four-stage clinical translation pipeline: 1) acoustic biomarker discovery, where acoustic features are shown to be representative of JIA disease activity, 2) JIA disease activity prediction using wearable sensing (KneeMS) and severity trend assessment from inactive to severe knees, 3) prospective longitudinal tracking of active JIA knee using KneeMS across follow-up clinical visits, 4) in-home monitoring where KneeMS will be deployed for self-assessment between clinical visits, where the wearable form-factor is essential. This knee-level prediction of JIA activity using clinically obtained labels makes this classifier a viable support for detecting JIA disease activity along with longitudinal follow-up that can be prospectively integrated in standard of care to provide quantitative data to clinicians during telemedicine visits. This can help detect any lack of progress in JIA activity and help in preventing progressive joint damage because of delays in follow-up visits.

Beyond clinical performance, the practical usability of KneeMS is crucial for its eventual deployment for at-home use. As a preliminary usability analysis, we collected participant discomfort scores on a scale of 0-10 (0 indicates no discomfort). These scores were collected during clinical visits of each participant and also for their follow-up visits (if any). Among the 42 collected scores from participants over their baseline and follow-up visits, participants reported minimal discomfort (median = 0, mean *= 0.55 ±  1.35*, n = 42). 79% of all participants reported no discomfort, while 90% reported a score of 2 or lower. These results show that the KneeMS was well tolerated in this pediatric cohort.

In its current form, KneeMS is a research prototype. It has not yet received regulatory clearance as a medical device. Therefore, the findings of this study should be interpreted as an early translational step towards a wearable, non-invasive knee monitoring system.

## IV. Limitations and Future Work

Although this work contributes to expanding a known KneeMS system for knee severity trend analysis for the JIA population, it faces limitations that will be addressed in future work. First, the number of samples for each knee severity category is different. The number of inactive knees far exceeds the number of each active knee category (mild, moderate, or severe), hence creating an imbalance in the dataset. In addition, the number of samples for longitudinal tracking of active/inactive knees is also limited, and hence would be future directions to pursue.

In this work, all the labels for whether the knee is active or inactive and the severity of activity were determined by a pediatric rheumatologist during clinical exams. For more conclusive evidence of knee activity, for future work, we plan to incorporate imaging biomarkers such as ultrasound to confirm the level of activity in JIA knees. In addition to it, we plan to include healthy controls in our future studies to make comparisons between active and inactive JIA knees with KAEs from healthy controls.

In addition to a machine learning pipeline presented in this paper, which relies heavily on feature engineering, we plan to explore explainable deep learning pipelines to use the waveforms and spectrograms to explore which parts of the training data were useful for making classifications for active versus inactive knees. This would expand on our current knowledge of the feature sets that have been used for making such predictions in previous work [31], [33].

In this JIA cohort, we asked every participant to express their discomfort on a scale of 0-10 while using the KneeMS device during their clinical visit. Although the majority of the participants expressed minimal discomfort, we would like to expand on the usability and patient adherence with additional surveys suited for at-home use of the KneeMS wearable sensor.

## V. Conclusion

In this work, we expand the capabilities of a previously validated KneeMS system by using it for the JIA population in a clinical environment, comparing it to a benchtop setup (Dytran). We expanded our previous work with KneeMS to validate it against a benchtop (Dytran) system using data collected from children with JIA [26]. We also expanded the feature set used to find correlations between the acoustic features of KneeMS and Dytran beyond the RMS feature [26], [39]. Of the 36 features used, we showed a high and significant Spearman correlation coefficient between six acoustic features i.e. RMS, spectral slope, spectral flux, energy, MFCC1, and Hjorth activity.

To demonstrate prospective clinical integration of KneeMS in standard of care in the future, we also showed that KneeMS can make knee-level predictions using a logistic regression classifier with an AUC-ROC of 0.77 with a significant permutation test (p = 0.005), in contrast to Dytran with an AUC-ROC of 0.64 with a non-significant permutation test (p = 0.0629), expanding on our previous work [44]. This shows that a wearable sensor like KneeMS can serve as a viable replacement for Dytran and make better knee-level predictions when trained on a cohort of active and inactive JIA knees. To further extend this comparison, we show that the KneeMS wearable data exhibited a significant trend in severity from inactive to severe knees (p = 0.001), similar to Dytran’s data (p = 0.047). In addition to this, we compared the prediction probability of active knees for our LR classifier for both KneeMS and Dytran for comparing which of these *P(A)*s move in a clinically significant direction. Based on this comparison, KneeMS’s *P(A)* moves in the relevant clinical direction (active to inactive or inactive to active) of JIA disease activity four out of 5 follow-up visits compared to one out of five for Dytran. This shows prospective use of the KneeMS device for future studies to examine longitudinal at-home use.

As an additional analysis for user perception of discomfort, we asked all our participants to rate user discomfort for using the KneeMS device during data collection during their clinical visits to CHOA. Among the 42 collected scores from participants over their baseline and follow-up visits, participants reported minimal discomfort (median = 0, mean *= 0.55 ±  1.35*, n = 42). 79% of all participants reported no discomfort, while 90% reported a score of 2 or lower. These results show that the KneeMS was well tolerated in this pediatric cohort.

## VI. Disclosures

O. T. Inan is a co-founder of Arthroba, Inc., a start-up focused on wearable joint health sensing technology.

## VII. Data Availability

Data will be made available on reasonable request to the corresponding author.

## Supplementary Materials

Supplementary Materials
