# Supplementary material for: KneeMS to Monitor Knee Acoustic Emissions for Juvenile Idiopathic Arthritis
Source: IEEE J Transl Eng Health Med. 2026 Aug 7;14:369–79. doi: 10.1109/JTEHM.2026.3721498 (PMC13724281; doi:10.1109/JTEHM.2026.3721498)
Supplement: Supplementary file 1 [file supp1-3721498.pdf]

Supplementary Materials

S1. Outlier Sensitivity:

We evaluated the change in Spearman's ρ before and after the removal of the FE cycle data beyond the ±1.96 SD in Table S1 and S2 for all features used. We also report the number of knees excluded for each feature from both the medial and lateral sides. From Table S1 and S2, it is clear that there is only minimal change in Spearman's ρ after the removal of outliers, which is consistent with our rational of choosing Spearman Correlation Coefficient instead of Pearson's correlation.

Table S1. Spearman correlation coefficients before and after removing Bland–Altman outliers (> ±1.96 SD), computed at the knee level (n = 68) for the medial side. All correlations are significant (p < 0.001).

Feature	ρ (all)	ρ (outliers removed)	Outliers (%)
RMS	0.79	0.78	2 (2.9%)
MFCC-1	0.79	0.87	5 (7.4%)
Spectral slope	0.78	0.77	2 (2.9%)
Energy	0.78	0.77	1 (1.5%)
Hjorth activity	0.75	0.73	2 (2.9%)
Spectral flux	0.74	0.73	2 (2.9%)
Spectral crest	0.62	0.75	4 (5.9%)
Hjorth complexity	0.58	0.71	3 (4.4%)
Peak-to-peak	0.55	0.56	4 (5.9%)
MFCC-7	0.51	0.63	5 (7.4%)
Hjorth mobility	0.49	0.59	4 (5.9%)
Spectral kurtosis	0.45	0.44	2 (2.9%)
Spectral concentration	0.44	0.47	4 (5.9%)
Spectral entropy	0.44	0.54	3 (4.4%)
MFCC-6	0.42	0.52	3 (4.4%)
Spectral skewness	0.39	0.41	3 (4.4%)
MFCC-5	0.38	0.47	2 (2.9%)
Waveform kurtosis	0.38	0.32	6 (8.8%)
TKEO max	0.38	0.38	2 (2.9%)
TKEO mean	0.36	0.34	1 (1.5%)
TKEO std	0.35	0.35	2 (2.9%)
MFCC-4	0.35	0.44	2 (2.9%)
MFCC-3	0.33	0.41	2 (2.9%)
MFCC-2	0.32	0.40	2 (2.9%)
Zero-crossing rate	0.30	0.44	5 (7.4%)
Spectral centroid	0.29	0.43	5 (7.4%)
Spectral flatness	0.24	0.35	4 (5.9%)
Spectral roll-off	0.22	0.36	5 (7.4%)
Spectral spread	0.16	0.28	3 (4.4%)
MFCC-12	0.10	0.14	1 (1.5%)
MFCC-11	0.10	0.14	1 (1.5%)
MFCC-13	0.07	0.13	2 (2.9%)

Feature	ρ (all)	ρ (outliers removed)	Outliers (%)
MFCC-10	0.07	0.12	1 (1.5%)
MFCC-8	0.07	0.07	5 (7.4%)
MFCC-9	0.02	0.06	1 (1.5%)
Waveform skewness	-0.32	-0.27	4 (5.9%)

Table S2. Spearman correlation coefficients before and after removing Bland–Altman outliers (> ±1.96 SD), computed at the knee level (n = 68) for the lateral side. All correlations are significant (p < 0.001).

Feature	ρ (all)	ρ (outliers removed)	Outliers (%)
MFCC-1	0.72	0.75	4 (5.9%)
Hjorth activity	0.71	0.71	1 (1.5%)
RMS	0.70	0.70	3 (4.4%)
Spectral flux	0.69	0.68	1 (1.5%)
Spectral slope	0.68	0.69	3 (4.4%)
Energy	0.67	0.65	2 (2.9%)
Peak-to-peak	0.59	0.65	4 (5.9%)
Hjorth complexity	0.58	0.67	5 (7.4%)
MFCC-7	0.57	0.61	1 (1.5%)
Spectral concentration	0.55	0.50	4 (5.9%)
MFCC-6	0.53	0.63	5 (7.4%)
TKEO mean	0.53	0.59	2 (2.9%)
Hjorth mobility	0.52	0.59	4 (5.9%)
TKEO std	0.51	0.51	1 (1.5%)
TKEO max	0.50	0.50	1 (1.5%)
MFCC-5	0.47	0.58	4 (5.9%)
Waveform kurtosis	0.47	0.37	5 (7.4%)
Spectral crest	0.47	0.49	2 (2.9%)
Spectral centroid	0.43	0.45	3 (4.4%)
Spectral entropy	0.43	0.49	2 (2.9%)
MFCC-4	0.43	0.53	4 (5.9%)
MFCC-3	0.40	0.50	4 (5.9%)
Spectral flatness	0.40	0.44	2 (2.9%)
MFCC-2	0.39	0.49	4 (5.9%)
Spectral skewness	0.38	0.42	4 (5.9%)
Spectral kurtosis	0.38	0.39	3 (4.4%)
Spectral roll-off	0.38	0.44	3 (4.4%)
Zero-crossing rate	0.37	0.41	2 (2.9%)
Spectral spread	0.30	0.37	2 (2.9%)
MFCC-8	0.23	0.36	4 (5.9%)
MFCC-12	0.19	0.18	3 (4.4%)

Feature	ρ (all)	ρ (outliers removed)	Outliers (%)
MFCC-11	0.19	0.21	4 (5.9%)
MFCC-13	0.18	0.18	5 (7.4%)
MFCC-10	0.16	0.23	5 (7.4%)
MFCC-9	0.15	0.23	5 (7.4%)
Waveform skewness	-0.22	-0.15	2 (2.9%)

Table S3. Spearman intercorrelation matrix among the six acoustic features for Dytran (lower triangle) and KneeMS (upper triangle), medial side (n = 68). All correlations significant (p < 0.001).

	RMS	MFCC1	Sp. Slope	Energy	Hjorth	Sp. Flux
**RMS**	—	0.966	−0.980	0.936	0.972	0.962
**MFCC1**	0.955	—	−0.967	0.850	0.904	0.897
**Sp. Slope**	−0.985	−0.939	—	−0.910	−0.945	−0.939
**Energy**	0.949	0.875	−0.935	—	0.972	0.962
**Hjorth**	0.986	0.923	−0.973	0.973	—	0.992
**Sp. Flux**	0.986	0.917	−0.975	0.973	0.998	—

S2. Window length and overlap sweep for Machine Learning

We experimented with different window lengths and overlaps during feature extraction to maximize the area under the curve of the receiver operating characteristics curve (AUC-ROC). Table S4 shows the combinations of these choices.

Table S4. AUC-ROC for different selection of window lengths and overlap %.

Window	Overlap %	KneeMS AUC-ROC	Dytran AUC-ROC
15	50	0.60	0.66
15	75	0.64	0.702
30	50	0.71	0.64
30	75	0.66	0.628
**50**	**50**	**0.77**	**0.64**
50	75	0.74	0.56
50	80	0.64	0.60

S3. Confusion Matrices for ML

As we can infer from Figure S1(a) and (b), KneeMS correctly identified substantially more active JIA knees than Dytran (19 versus 13 of 28 knees), along with more inactive knees (38 vs 34 of 47 knees), indicating that

the wearable's advantage lay primarily in its sensitivity to active disease. KneeMS achieved a sensitivity of 0.679, specificity of 0.809, and balanced accuracy of 0.74, compared with a sensitivity of 0.46, specificity of 0.723, and balanced accuracy of 0.59 for Dytran.

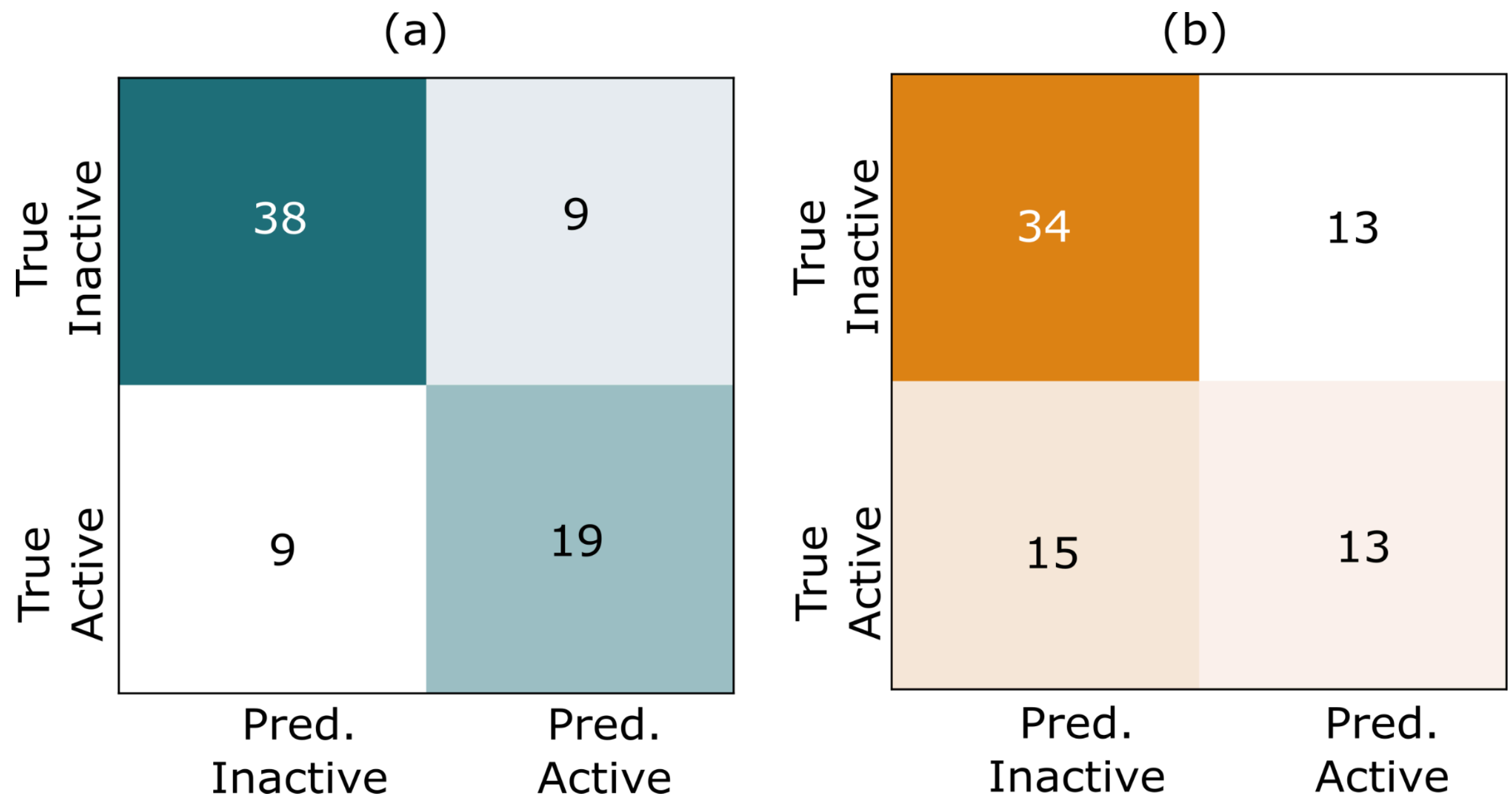

Figure S1. Confusion matrices for (a) KneeMS-based LR pipeline, (b) Dytran-based LR pipeline.
